# Fecal Molecular Markers for Colorectal Cancer Screening

**DOI:** 10.1155/2012/184343

**Published:** 2011-11-17

**Authors:** Rani Kanthan, Jenna-Lynn Senger, Selliah Chandra Kanthan

**Affiliations:** ^1^Department of Pathology and Laboratory Medicine, Royal University Hospital, University of Saskatchewan, Saskatoon, SK, Canada S7N 0W8; ^2^Department of Surgery, University of Saskatchewan, Saskatoon, SK, Canada S7N 0W8

## Abstract

Despite multiple screening techniques, including colonoscopy, flexible sigmoidoscopy, radiological imaging, and fecal occult blood testing, colorectal cancer remains a leading cause of death. As these techniques improve, their sensitivity to detect malignant lesions is increasing; however, detection of precursor lesions remains problematic and has generated a lack of general acceptance for their widespread usage. Early detection by an accurate, noninvasive, cost-effective, simple-to-use screening technique is central to decreasing the incidence and mortality of this disease. Recent advances in the development of molecular markers in faecal specimens are encouraging for its use as a screening tool. Genetic mutations and epigenetic alterations that result from the carcinogenetic process can be detected by coprocytobiology in the colonocytes exfoliated from the lesion into the fecal matter. These markers have shown promising sensitivity and specificity in the detection of both malignant and premalignant lesions and are gaining popularity as a noninvasive technique that is representative of the entire colon. In this paper, we summarize the genetic and epigenetic fecal molecular markers that have been identified as potential targets in the screening of colorectal cancer.

## 1. Introduction

In Canada, despite increased awareness with improved screening techniques, colorectal cancer (CRC) remains the second leading cause of death from cancer in both men and women [1]. When detected at stage I or II, surgical cure rates approach 90% and 75%, respectively [2, 3]; however, detection is often postponed until patients become symptomatic [4], which may not occur until 2-3 years later, by which time the lesion is often of high grade [5]. As such, detection of precancerous lesions and early CRC is vital to achieving the ultimate goal of screening: decreased incidence and mortality due to CRC. The ideal screening technique should be (a) able to detect disease at a curable stage, (b) both highly sensitive and specific, (c) able to elicit a high participation rate, (d) affordable, (e) safe for the patient and the physician, (f) more beneficial than the adverse effects, and (g) easy to perform [6, 7]. Current screening techniques do not accomplish these noble goals.

Colonoscopy is currently considered the “gold standard” of CRC screening; however, despite recommendations, less than 60% of eligible individuals over the age of 50 have undergone this test [8]. Factors including patient discomfort, invasiveness, embarrassment, high cost, and considerable expertise and equipment required may all limit the appeal of this screening technique [8, 9]. Flexible sigmoidoscopy (FS) has shown promise, identifying 50–70% of advanced distal lesions [10]; however, approximately 1/3 of the neoplasms are too proximal for detection [5] and the procedure is invasive and cumbersome for patients [11]. Noninvasive method of fecal occult blood testing (FOBT) has gained popularity as a detection tool for CRC. There are two techniques for the detection of occult blood hemoglobin: (a) chemical/enzymatic FOBT by reacting with the peroxidase of the heme group, often relying on guaiac as a reagent, and (b) immunochemical/immunological FOBT that uses antibodies against human globin [12, 13]. This technique has reduced CRC mortality by 15–33% [14, 15]; however, it is limited as it (a) may detect bleeding from any site including the stomach or small bowel, (b) may falsely react with plant peroxidases or heme in red meat, and (c) can only detect actively bleeding lesions. As such, sensitivity to precursor lesions such as adenomas lies in the low 10–20% range [10]. Additionally, false-negative and false-positive results frequently occur. As such, a screening technique that combines high sensitivity and specificity for adenomas and early-stage cancer, minimal invasiveness, safety, affordability, and acceptability by patients and physicians is required [16]. One of the significant advantages of colonoscopy is that, in addition to detecting the malignant tumours, adenomas and other benign precursor lesions can be detected and removed. This will not only reduce the mortality from colorectal carcinoma but also decrease the incidence of this disease. This is a noteworthy advantage in comparison with FOBT that cannot effectively reduce the incidence of colorectal disease. 

In 1989 it was first observed that viable gastrointestinal cells could be recovered from human stools and thus began the science of coprocytobiology [17]. Since then, the understanding of the natural history of CRC and its carcinogenic pathway has improved. With this increased understanding it is expected, therefore, that this improved knowledge should translate to better screening techniques that are more accurate and acceptable while minimizing their invasiveness [5]. Detection of molecular markers in fecal specimens is a promising screening technique. This noninvasive test has shown higher levels of specificity and sensitivity for the detection of precancerous and cancerous colorectal lesions and is associated with greater patient compliance. It has additionally been suggested that sensitivity rates of many of these molecular markers may increase with repeated testing within a well-defined screening program [18, 19]. The continued evolution in the studies of genomics, transcriptomics, proteomics, and metabolomics allows for the continued identification of potential cost-effective, safe, and simple molecular markers [20]. Comparisons between these tests can be difficult due to differences in methodologies and study populations [18]. In this paper, we summarize the single- and multipanel molecular markers that have been described in the recent literature.

## 2. Materials and Methods

Using PubMed and Google Scholar, an English literature search was performed using the text phrases “colorectal” and “molecular marker” published within the past 10 years (since 2000). Articles were restricted to fecal/stool specimens. The PubMed search listed 85 articles while the Google Scholar listed 6470 entries. The PubMed “Related Articles” feature identified an additional 45 relevant articles. Reference lists from these manuscripts were reviewed, and secondary articles were read and analyzed. Articles chosen were limited to fecal genetic and epigenetic markers. A total of approximately 150 articles were read, and 87 of these were finally included in this paper. We begin this paper with a brief introduction of the current understanding of carcinogenesis pathways in the development of CRC.

## 3. Development of CRC

An understanding of the carcinogenic pathway leading to the development of CRC is necessary to comprehend the use of molecular markers as a screening tool. As in the general population the majority of cancers (85%) are sporadic [6], and early identification of precursor lesions may provide the opportunity to intervene in the carcinogenic process at a curable stage. Early detection is central to decreasing mortality and morbidity. These malignancies are believed to develop due to an accumulation of mutations in oncogenes, tumour suppressor genes, and DNA mismatch repair genes [21]. Traditionally, three separate pathways in the development of CRC have been described.

- *The Chromosomal Instability Pathway* is the most common sequence in the development of CRC, caused by whole or partial chromosomal loss/mutation [22]. It is hypothesized that a step-wise accumulation of mutations in a variety of genes, including oncogenes and tumour suppressor genes, results in abnormal cells that have a greater propensity for proliferation and growth [6]. The proposed process, with genes typically implicated, includes sporadic mutation in one allele of a gene (proposed *APC* gene on chromosome 5q) that results in the formation of dysplastic crypts [23, 24]. Genomic instability incurs a growth advantage to mutated cell lines permitting clonal expansion [10]. Mutation to the *K-ras* gene on chromosome 12 causes progression from an early-type adenoma to an intermediate-  and then late-type adenoma. Mutations to *DCC* gene on chromosome 18 may enhance cell growth and spread. Finally, a *TP53* mutation on chromosome 17p in large adenomas with severe dysplasia promotes conversion to a carcinoma [22, 23]. It has been additionally suggested that hyperplastic polyps, like adenomas, may develop into cancerous lesions through a similar step-wise progression [25] via a serrated adenoma intermediate [26]. Serrated adenomas and serrated hyperplastic polyps are reported to have high rates of microsatellite instability and lower rates of mutations in *APC, p53*, and *K-ras* [27]. It is suggested that 20–30% of CRCs are derived from serrated polyps rather than adenomas. These occur in a more elderly population, are frequently right sided, endoscopically less obvious, and have a faster growth potential [7].
- *The microsatellite instability pathway* appears to occur in 12–15% of all CRCs. In these patients, a loss of the DNA mismatch repair system results predominantly in mutations to large poly-A-regions (big adenine tracts—BAT) and CA-repeats [10]. Additionally, resulting mutations from inactivation of this system could affect all microsatellites, not only BAT and CA-repeats. This pathway is explained further in Section 7.1.
- *The third pattern of development of CRC* includes the 2-3% that do not fit into the above two categories [10]. The epigenetic process of methylation of the promoter regions of genes is common in neoplasia and causes silencing of the gene, that is, being “turned off” [22]. As such, methylation of tumour suppressor genes may promote the development of cellular proliferation and lead to tumour development. Methylation of CpG islands is one of the primary epigenetic changes involved in the pathogenesis of CRC that is detectable in fecal specimens [2]. Two of these methylation pathways of CRC include those with rare methylation (CIMP−) and those with aberrant methylation of multiple genes (CIMP+). This is further explained in Section 7.3 under the “Epigenetic Changes.”

The events of these pathways transpire over an extended period of time, with conservative estimates of 5–10 years being required for the development of CRC [26]. This interval therefore provides a window of opportunity to detect the adenomatous or early CRC lesions while still at an early curable stage. Determining which adenomas will become carcinomatous can be difficult, as over 50% of individuals will develop adenomatous lesions over their lifetime while only 6% will have malignant transformations [7]. Differentiation between these two lesions to identify and remove precancerous polyps is a key feature of the optimal screening exam to prevent CRC-related death [28]. Certain characteristics infer a greater risk of progression. Factors including severe dysplasia, a villous histological type, large size (≥1 cm), and the patient's age are risk indicators of potential malignant transformation [26, 28]. Identification of these features in an adenomatous polyp and its subsequent removal may reduce the incidence of CRC and mortality in high-risk groups [29]. Though many molecular tests have achieved a relatively high sensitivity for cancerous lesions, tests to detect precancerous lesions such as high-risk adenomas have not been adequately studied [20]. Further, current screening continues to be ineffective, as only 37% of CRCs are diagnosed while the cancer is localized. Such inefficiencies in screening may be due to suboptimal sensitivities, low patient acceptability, or high resource demands that limit availability [29]. 

## 4. Molecular Markers

The ideal biomarker for the detection of CRC and premalignant lesions would be (a) consistently positive in the presence of “screen-relevant neoplasia” and negative in the absence, (b) stable despite fecal toxicities, (c) easily recoverable from the stool, and (d) reproducibly assayed [30]. The multiple genetic events associated with the development of CRC coupled with the long-time interval between the initial adenomatous/polypoid event and the cancerous lesion suggest a role for molecular markers in early detection. These specific changes occurring within the DNA, RNA, and proteins may potentially serve as biomarkers and be used as screening, diagnosis, and as predictive and prognostic markers in CRC. These genetic alterations may be due to gene mutations, gene amplification, aberrant DNA methylation, or chromatin modifications [20]. The ultimate goal of the use of molecular markers is a biomarker panel to detect carriers of early CRC or precursor lesions in order to reduce the incidence and mortality of this highly prevalent cancer. Molecular markers must be both highly sensitive and specific. Methylation of markers such as estrogen-receptor and insulin-like growth factor II may be detected in patients with CRC; however, such methylation is additionally common in aging colon and is thus an unattractive marker [15]. An effective marker for detection should be regularly released from the tumour/precancerous lesion, withstand metabolic degradation, and be readily retrievable and measurable from the medium collected [7]. 

Such markers may be found directly in the tissue; however, retrieval requires an invasive procedure. More recently, assays have been developed that detect genetic materials shed from CRC into faecal specimens. Sidransky et al., in 1992, were the first to use fecal samples to detect CRC by testing for mutations in the *K-ras* gene [31]. Over the past thirty years, a wide array of single genetic and epigenetic changes that are detectable within fecal samples have been identified. Concurrently, multimarker panels have been proposed. The first multimarker panel was published eight years later (2000) when Ahlquist et al. published a trial with 15 point-mutations on *K-ras, p53, APC, BAT-26* and long-DNA (L-DNA) [32]. Many of the single and multi-panel genes that have been identified as potential molecular markers are herein summarized. 

## 5. The Process of Fecal-Based DNA Testing

 Colonocytes that are present on the surface of the colonic mucosa are continuously shed into the lumen of the colon and excreted as a normal component of stool [6, 17]. These cells are shed from the lower crypts at a rate of at least 10^10^ cells per day, each with a lifespan of 3-4 days [33]. The renewal turnover is 1% per hour, and within four days the entire colonic mucosa is renewed [5]. In the 19th century it was first observed that exfoliated cells in colonic washings could be used for the diagnosis of CRC. Current advances in coprocytobiology [17] understand that, in the normal colon, exfoliation is triggered by apoptosis or anoikis (involution) when separated from the basement membrane. In malignant lesions, however, genetic and/or epigenetic changes prevent this destruction of colonocytes, allowing them to survive within the stool. This may be due to increased cellular proliferation or reduced cell-cell or cell-basement membrane adhesions [7]. Additionally, the number of colonocytes exfoliated from malignant lesions is 4-5-fold greater than from normal tissue [5]. As such, tumour markers present within these cells are preserved. Within the stool are bile acids and cytolytic compounds that may lyse colonocytes and expose the DNA to metabolically active microflora and fecal proteases that may destroy the material and catabolize potential tumour markers [7]. To prevent this natural occurrence, the addition of a DNA-stabilizing buffer immediately afterdefecation has been reported to prevent DNA degradation for several days [34]. DNAase inhibitors in these buffers prevent degradation during transportation and storage of these specimens until they can be examined [7]. The somatic cell sampling and recovery (SCSR) process involves the isolation of exfoliated colonocytes from a small sample of stool that can be collected and transported in a unique medium at suitable temperatures to provide cells for the detection of a number of biomarkers [17]. Such cells with mutations in key genes or alterations in protein products can then be analyzed with molecular biology or biochemical techniques [21].

To begin the analysis, abnormal genetic material must then be separated from normal human DNA and bacterial DNA prior to amplification and testing for molecular markers [29]. Human genetic material within fecal specimens is sparse, with a median concentration of 309 ng/g stool (ranging 5–21 115 ng/g stool) [35]. This accounts for only 0.01% of the total genetic material in DNA, the remaining portion including genetic materials from colorectal microbial flora, eukaryotic parasites, and undigested dietary remains [7]. As such, materials recovered from stool must be enriched to retrieve gene sequences for PCR analysis. After amplification genetic molecular markers can be detected. The wide variety of methodologies for the detection of these markers is beyond the scope of this paper and will not be discussed.

 A study examining the optimal number of fecal specimens for molecular screening exams found no additional benefit for more than one specimen per patient, with a 93% concordance between the initial results and all subsequent analyses as no additional mutations were detected on second or third screenings [36]. Due to the heterogeneity of fecal matter, it is, however, important that screening includes samples from the entire stool. Depending on the site of the lesion, markers may be present in different parts of the stool, as left-sided tumours tend to have markers represented on the surface whereas in the unformed feces from right colon colonocytes may be found throughout the sample [7].

## 6. Single Genetic Markers

The four most commonly studied genes for faecal CRC include the *KRAS, TP53, APC,* and *DCC* genes. Detection of *DCC* mutations in fecal specimens is not well studied; however, *KRAS, TP53, *and* APC *are well reported in the English literature, as, for example, Calistri's study reports that *K-ras* and *p53* were equally altered in 35% of CRC patients and *APC* mutations were reported in 13% [37]. These genes are now discussed in detail. 

### 6.1. *KRAS* Gene

The *KRAS* gene is located on the short arm of chromosome 12 and encodes the *K-ras*guanosine- triphosphate- (GTP-) binding protein with a role in signal transduction for regulation of proliferation and differentiation [6]. Normally the *K-ras* protein hydrolyzes GTP, thereby inactivating the ras protein [6, 38]. This was the first molecular marker to be studied in fecal specimens nearly 30 years ago [31]. Mutations to this gene are more common in lesions of the distal colon [18]. Activating mutations of *KRAS* may result in uncontrolled cellular proliferation and resistance to EGFR-targeted therapies [39]. Only one copy of this gene needs to be mutated to cause uncontrolled cell growth, as *K-ras* is a signal transducer [38]. The majority (70–80%) of these mutations are on codon 12, although they may occur on codons 13 and 61. With fecal specimens, *K-ras* mutations have been detected in 35–42% of CRCs and approximately 50% of adenomas larger than 1 cm [6]. This marker remains nonspecific as it has been detected in stool from morphologically normal colonic mucosa, self-limiting hyperplastic polyps, and nondysplastic aberrant crypt foci [15]. Additionally, this marker is nonspecific for CRC, as its fecal presence may be positive in benign, unrelated pathology such as pancreatic hyperplasia [5].  Table 1 summarizes the sensitivity and specificity of *KRAS* studies as available in the reviewed literature which are briefly discussed below.

**(i) Zhang et al. (2011) [39]**

A chip-based temperature gradient capillary electrophoresis (TGCE) technique was used to detect mutations in *K-ras* among stool specimens from 30 CRC patients and 15 normal-controls. A total of 17/30 CRC patients demonstrated *K-ras* mutations (57%), and only 1/15 controls had the mutation, thus yielding a specificity of 93%.

**(ii) Chien et al. (2007) [40]**

This study explored the role of this molecular marker in the identification of CRC. *K-ras* codon 12 mutations were identified using reverse transcription-polymerase chain reaction (RT-PCR) and amplified restricted fragment length polymorphism analysis, feces from 5% of “normal” control, and 41% of CRCs. These mutations were significantly associated with a younger patient age.

**(iii) Øgreid and Hamre (2007) [27]**

In this paper, *K-ras* mutations were detected in a stool sample 18 months prior to its endoscopic identification.

**(iv) Rengucci et al. (2001) [41]**

Stool specimens retrieved from 46 patients with CRC showed 6 to be positive for *K-ras* mutations: 2 in exon 2 and 4 in exon 1. One-third of tissue samples were positive for this mutation.

**(v) Notarnicola et al. (2000) [42]**

*K-ras* mutations were detected in fecal samples from 26 CRC patients using PCR amplification and restriction enzyme analysis. Fecal *K-ras *mutations were detected in 26.9% of cases.

**(vi) Smith-Ravin et al. (1995) [43]**

11 patients with sporadic CRC provided stool samples to be analyzed for the *ras* mutation with a nonradioactive, allele-specific mismatch method. Approximately half of the stool samples were positive for the mutation.

The use of the *KRAS* gene as a molecular marker has not been supported in CRC screening by several studies [44, 45]. Though this gene can detect some individuals with CRC, it is often ineffective at identifying individuals who are at an increased risk of developing this cancer based on preneoplastic lesions. It is not associated with risk factors or with the identification of high-risk individuals [44]. This marker is a common component of multitargeted assays, as described later.

### 6.2. *TP53* Gene

As elucidated in the adenoma-carcinoma pathway, mutations in the gene *TP53* most commonly occur in the later stages of CRC. This gene is located on the short arm of chromosome 17 and encodes the gene p53, a well-studied player in many forms of human carcinogenesis. Mutations may occur at exons 5, 6, 7, or 8 [6, 45]. At mutation, the p53 protein is deregulated, resulting in an increased genomic instability and malignant progression. On fecal screening, such mutations have been identified in 50–70% of CRCs [5], up to 64% of severely dysplastic polyps [10], and 4–26% of adenomas [6]. Conflicting results regarding the detection rate of this mutation exists in the literature as seen below.

**(i) Rengucci et al. (2001) [41]**

Stool and tissue samples were received from 46 patients with CRC. Though in 37% of tissue samples (*n* = 17) this marker was present, in fecal samples only 3 patients had mutations, two found on exon 6 and one in exon 8.

**(ii) Notarnicola et al. (2000) [42]**

*p53* mutations were detected in fecal samples from 26 CRC patients using PCR amplification and single-strand conformation polymorphism. Mutations were detected in 50% of cases.

### 6.3. *APC* Gene

The *adenomatous polyposis coli* (*APC*) gene is found on chromosome 5q21 and encodes the APC “gatekeeper” protein that is responsible for the regulation of *β*-catenin, an inductor of growth-promoting genes in the *Wnt* signalling pathway [6]. Additional roles of APC include regulation of cell adhesion, interaction with microtubules for cell migration, and blockage of cell cycle [46] Mutations to the *APC* gene occur early in the adenoma-carcinoma sequence and in fecal screening are reported to be detected in 20–82% of adenomas and 52–60% of CRCs [6]. Unlike *KRAS* and *TP53*, mutations are not limited to a small region and can occur anywhere along the first 1600 codons of the gene, [5] though 83% occur within the first part of the sequence [10]. Such mutations occur in both inherited and sporadic forms of CRC [12]. A sample of the results of detection of this marker in a couple of studies are listed below.

**(i) Suceveanu et al. (2008) [47]**

In this study (*n* = 200), 15 exons of the *APC* gene were analyzed for mutations, which were identified in exons 4 (9 patients), 9 (1 patient), 13 (6 patients), and 15c (5 patients) with none occurring in exons 5, 7, 8, 10, 12, and 15.

**(ii) Traverso et al. (2002) [48]**

DNA was purified from stool samples of 28 patients with CRC, 18 with adenoma (≥1 cm), and 28 normal-controls. Samples were screened for *APC* mutations with digital protein truncation. Mutations were present in 57% patients with neoplasia (26/46) and in none of the controls.

### 6.4. Additional Gene Mutations

Additional gene mutations in fecal specimens that have been explored in the literature include the following.

**(i) DCC**

The deleted in colon cancer (*DCC*) gene is found on the long arm of chromosome 18 and under normal circumstances maintains cell-cell adhesions. Though well-studied in tissue specimens, its expression in faecal samples remains poorly researched and understood. In one study, on fecal analysis, mutations were reported in 70% of CRCs, 11–13% of small adenomas, and up to 60% of adenomas with malignant foci enhancing cell growth and metastatic spread [6]. Further research in this area is required to elucidate the potential role of *DCC* as a molecular marker.

**(ii) RPL19 [49]**

The gene *RPL19* is responsible for encoding the ribosomal protein L19. In a study of fecal matter from 44 CRC patients, 15 controls, and 11 colonic cell lines, a quantitative real-time reverse transcription PCR detected 7/24 patients with late-stage CRC expressed 2-times more RPL19 in colonic tumour tissue than normal, and the mean fecal RPL19 mRNA levels of late-staged patients were also higher than the controls. The authors concluded that the RPL19 protein is associated with increased expression in advanced CRC patients and this is detectable in fecal matter.

**(iii) COL11A1 [47]**

The COL11A1 gene encodes the collagen *α*-1 chain protein. Mutations to this gene in CRC are not well studied in the feces. In Suceveanu's study (*n* = 200), fecal samples underwent denaturing gradient polyacrylamide gel electrophoresis. Exons 16, 38, 41, 54, 55, 56, and 57 were studied. In exons 16, 38, and 41 no mutations were detected; however, a displaced band pattern was detected in 6 cases for the exons 54, 55, 56, and 57.

**(iv) COX-2, Matrix-Metalloproteinase-7 mRNA [7]**

Fecal analysis of mutated COX-2 yielded a specificity of 100% and sensitivity of 87%. Analysis of matrix metalloproteinase-7 mRNA was detected in 65% of patients. 90% of CRCs can be detected by these two markers combined.

**(v) C-myc p64, c-myc p67, and c-erbB2 [50]**

Colonocytes were isolated in stool samples from 15 patients with CRC and 15 normal-controls, and using reverse transcriptase PCR the expressions of c-myc p64, c-myc p67, and c-erbb-2 were evaluated separately and in combination. C-myc p64 was expressed in 78.5% of CRC patients and only 13.3% of the control (sensitivity 86.7%). C-myc p67 was detected in 78.6% of CRC patients and 13.3% of controls (sensitivity 86.7%), and c-erbb-2 showed no significant difference in mRNA expression between CRC and controls. In a panel combination assay, a sensitivity of 64% was found, with 100% specificity.

## 7. Introduction to Epigenetic Changes

### 7.1. Fecal Microsatellite Instability

Microsatellite instability (MSI) occurs when short stretches of DNA sequences with a tandem repeating pattern (a microsatellite) undergo a change in length due to a loss of function of at least one of the six mismatch repair genes (*MLH1, MSH2, PMS1 PMS2, MSH3, MSH6) *resulting in an accumulation of mutations and errors of replication causing lengthening or shortening of the microsatellite [6, 12, 22]. These errors in replication can be identified with microsatellite marker alleles, nucleotide sequences present in tumour DNA but absent from normal genetic material [46]. In sporadic CRC, MSI is most often associated with epigenetic silencing of the mismatch repair gene *MLH1 *[12]. MSI commonly affects genes with microsatellites in their coding region [22]. This instability is commonly detected in patients with hereditary nonpolyposis CRC (>90% in CRCs and 80% adenomas), but less frequent in sporadic CRCs (15%) and adenomas (5%) [5, 22]. MSI has been shown to confer a better prognosis compared to stage-matched stable tumours [12]. The most common fecal marker of MSI used is Big Adenine Tract-26 (*BAT-26*), a locus of 26 repeated adenine nucleotides located in the *MSH-2* mismatch repair gene [5, 22, 24]. Among right-sided cancers proximal to the splenic flexure, BAT-26 is a feature in 30–40% [45]. This fecal marker alone has a sensitivity of 40% for proximal CRCs [5], and is more commonly used as part of a multitarget panel. 

### 7.2. Long DNA

Long DNAs (L-DNA) are genetic sequences as long as 1800–2400 base pairs that can be identified in faecal specimens and used to detect CRC [6]. By corpocytobiology techniques [17], in colonocytes exfoliated into the lumen from “normal” colon, nuclear endonucleases are activated during apoptosis, disintegrating cellular DNA into fragments of 180–200 base pair. In CRC, cells are nonapoptotic; therefore, when they are shed from the tumour, they are not subjected to this degeneration and can be retrieved from stool samples [22]. Using the fluorescence L-DNA method, authors have amplified stool DNA and found that the average value for CRC was 64 ng (range 0–731 ng) compared to 0 ng (range 0–246 ng) in the healthy control. This study further found that, with a cut-off value of 25 ng, sensitivity of 79% and specificity of 89% could be achieved in fecal testing [51]. This is in concordance with another study that purified DNA from stool samples and found DNA fragments from patients with CRC to have a higher molecular weight than those from the control (>18/24 possible bands detected) [52]. In terms of its use as a fecal screening tool, L-DNA is often unstable during storage; however, the addition of a buffer with a DNAase inhibitor appears to remedy this problem. This was explored by Zou et al. using a real-time *Alu* PCR assay for quantifying faecal L-DNA. The authors found an average 75% drop in L-DNA levels in nonbuffered fecal specimens within the first day, yet, with the addition of an EDTA buffer, the integrity of the DNA was preserved. L-DNA is not specific for CRC, as its presence in stool specimens may be due to cancer of the upper gastrointestinal tract or from inflammatory bowel disease [53]. In Abbaszadegan's study, L-DNA was detected in 64% of stool samples from patients with CRC (*n* = 45) with a specificity of 95% (control *n* = 20) [54].

### 7.3. Methylation Markers

One of the primary epigenetic changes involved in the pathogenesis of CRC that can be detected in fecal specimens is the methylation of CpG islands [2]. CRC can be divided into two subtypes: those with rare methylation (termed CIMP−) and those with aberrant methylation of multiple genes (CIMP+). CIMP or “CpG island methylator phenotype” is increasingly recognized as a clinically and etiologically distinct group with its own epidemiology, histology, and molecular features. CIMP-positive CRCs commonly have a more frequently mutated *K-ras* gene but fewer mutations to *TP53* [55–57].

 In this process, a methyl group is enzymatically transferred from the methyl donor S-adenosylmethionine to the carbon-5 position of the cytosine [26]. Hypermethylation of these cytosine residues is responsible for their transcriptional inactivation [58]. They are often identified within untranslated first exons or the promoter regions of genes responsible for regulating cellular proliferation, apoptosis, and DNA repair [2, 29]. Though there may be hundreds of hypermethylated genes, only a select few play a significantly functional role in the development of CRC and therefore are potential molecular markers [29].

By using methyl-binding domain protein columns to capture methylated DNA, sensitivity has been shown to be markedly increased without negatively affecting specificity [59]. Detection of genetic mutations can be challenging, as a single gene can be mutationally inactivated through multiple mechanisms or mutated at varying positions. By contrast, in hypermethylation, it is often identical residues in the regulatory regions of the particular gene that are targeted by cancers, thus facilitating screening test design [60]. DNA methylation patterns have been detected during early stages of tumourigenesis at the same or greater frequency as genetic mutations. In the majority of CRC's, specific methylated genes are attractive candidates for molecular detection in stool samples [7]. Some authors have suggested that the high cost and relatively low sensitivity associated with detection of DNA mutations preclude its usefulness in population-based testing; however, methylation-based testing has been suggested to be sensitive in the detection of CRC and an increasing number of specific markers representing epigenetic signatures are being identified. Such fecal markers are attractive screening tools due to their high prevalence in early-stage neoplasia and predictability as assay targets on gene promoter regions [35]. These fecal screening tests may additionally be used as early disease markers, prognostic indicators, and predictors of therapy response [61]. Genome-wide analyses for the identification of epigenetic target genes have provided an extensive list of marker candidates, from which a large number have been studied in detail in fecal samples [11]. 

## 8. Single Epigenetic Markers

### 8.1. *SFRP2*

The *SFRP2 *gene is responsible for encoding the secreted frizzled-related protein 2 (SFRP2). It is one of the *SFRP* tumour suppressor genes responsible for glycoprotein secretion to inhibit the Wnt tumourigenic pathway [62]. As such, when *SFRP* genes are silenced, the Wnt/*β*-catenin signalling pathway is activated. This gene contains a region rich in cysteine residues that can be methylated in CRC, and thus used as a fecal molecular marker. Hypermethylation of this gene in stool-based screening has been detected in 77–90% of CRCs with amplifiable DNA. Specificity is lacking as 23% of the “healthy” controls were found to have methylation at this locus as well. This might be explained by the understanding that foci of premalignant aberrant crypts, which are undetectable on a routine colonoscopy, may be hypermethylated at this site [15]. A number of studies have investigated the methylation of this gene as a potential fecal molecular marker. The summarized results of the sensitivity and specificity of some of these studies are in Table 2 and briefly discussed below.

**(i) Tang et al. (2011) [63]**

Stool, serum, and tissue samples from 169 CRC patients, 63 with advanced adenoma, 46 with nonadenomatous polyps, and 30 “normals” were retrieved and human DNA was analyzed with MS-PCR to detect methylation of *SFRP2*. In CRCs, the sensitivity of tissue analysis was the highest (88.2%) followed closely by stool (84%) and serum DNA (66.9%). Methylation of *SFRP* was less pronounced in adenomas, with sensitivities at 65.1%, 46%, and 6.4%, respectively. For the controls/normal, detection was low, at 0%, 6.7%, and 0% in tissue, stool, and serum. Overall the specificity of this marker in tissue samples was 34.9%, in stool was 54%, and in serum was 93.7%.

**(ii) Wang and Tang (2008) [64]**

Patients with CRC (*n* = 69), adenoma ≥1 cm (*n* = 34), hyperplastic polyps (*n* = 26), and “normal” (*n* = 30) provided stool and tissue samples. Fluorescence-based real-time PCR was used to analyze the *SFRP2* gene with clinicopathological correlation. Sensitivity of fecal specimens was 87.0% (60/69) for CRC, 61.8% (21/34) for adenoma, and 42.3% (11/26) for hyperplastic polyps. Two “normal” patients had hypermethylation of *SFRP2* in their fecal specimens. Hypermethylation of this gene was not significantly associated with sex, age, tumour stage, site, lymph node status, or histological grade.

**(iii) Oberwalder et al. (2008) [26]**

Methylation status by MethyLight was compared between patients with colorectal polyps and those with a negative colonoscopy. None of the healthy controls were found to have methylation of this gene. 33% of hyperplastic polyps and 46% of adenomas were positive for methylation. These authors concluded that *SFRP2* was the most sensitive fecal-based single DNA-based molecular marker for the identification of CRC.

**(iv) Huang et al. (2007) [62]**

Methylation of *SFRP2* was detected in 94.2%, 52.4%, 37.5%, and 16.7% of patients with CRC, adenoma, hyperplastic polyps, and ulcerative colitis, respectively. In the control, “healthy” subjects, only 1/24 stool analyses revealed methylation of this gene.

**(v) Müller et al. (2004) [65]**

A sensitivity of 90% and a specificity of 77% were reported for *SFRP2* in this study individually examining the methylation status of 44 genes. When repeated in a fecal DNA-independent test set (*n* = 26), a sensitivity and specificity of both 77% were found. The authors concluded methylation of *SFRP2* to be a sensitive single DNA-based marker to identify CRC.

### 8.2. *Vimentin*

The *Vimentin* gene is responsible for coding an intermediate filament protein constituent that normally is not expressed in colonic epithelium [2]. Normally *Vimentin* is expressed in mesenchymal-derived cells including fibroblasts, macrophages, smooth muscle cells, and endothelial cells [66]. This gene is usually neither methylated nor transcriptionally active in normal epithelial crypt cells [55]. A total of only 4 ng of human DNA is required within a fecal specimen for the detection of methylated *Vimentin *when captured using methyl-binding domain (MBD) protein and chelating the protein into a nickel-agarose matrix in a chromatography column [35]. The following studies highlight the usefulness of this fecal marker.

**(i) Ned et al. (2011) [66]**

A commercially available fecal DNA test named *ColoSure* has been developed that detects methylation of the *Vimentin* gene. There remains no published data on the sensitivity or specificity of this test.

**(ii) Baek et al. (2009) [29]**

60 individuals with CRC, 52 with adenomas, and 37 normal-controls were investigated for *Vimentin* methylation using methylation-specific PCR (MS-PCR) with specially designed primers. A sensitivity of 38.3% for CRC and 15.4% for adenomas was found.

**(iii) Itzkowitz et al. (2007) [34]**

In the buffered stool samples from 40 patients with CRC and 122 normal-controls, these authors found that detection of *Vimentin* methylation alone resulted in 72.5% sensitivity and 86.9% specificity.

**(iv) Chen et al. (2005) [2]**

A study focusing on fecal detection of *Vimentin* hypermethylation was conducted using MS-PCR in 94 patients with CRC and 198 normal-controls. This study used MS-PCR to determine that the methylation status of vimentin exon 1 in fecal DNA from CRC patients and “healthy” controls was compared. An overall sensitivity of 46% was documented (43/94) and specificity of 90% (10% of fecal DNA samples from the control were positive for vimentin methylation).

### 8.3. *MGMT, MLH-1,* and *CDKN2A*

The genes *MGMT, MLH-1* and *CKDN2A* have been proposed as potential fecal molecular markers. In one study, stool from “healthy” (*n* = 37), adenomas (*n* = 52), and CRC (*n* = 60) was investigated for methylation of *methylguanine DNA methyltransferase (MGMT)* and* human mut I homolog-1 (hMLH-1).* Methylation was detected in fecal samples from 51.7% and 30.0% of those with CRC and 36% and 11% of those with adenomas. With the addition of *Vimentin* methylation detection, a combined sensitivity for CRC was 75%, adenoma was 59.6%, and a specificity was 86.5% [29]. 

A second study assessed the methylation status of *MGMT, MLH-1,* and *CDKN2A *in colonic adenomas and hyperplastic polyps. The *MGMT, CDKN2A*, and *MLH-1* genes were methylated in 48%, 31%, and 0% of adenomas and 16%, 27%, and 10% of those with no detectable pathologies. Tubulovillous and villous adenomas were more frequently methylated compared with tubular adenomas. Adenomas with at least one methylation were typically larger than lesions without methylation (15.6 mm versus 7.0 mm). These findings suggest that hypermethylation of these genes may contribute to progression of polyps to carcinoma [25].

Additional epigenetic alterations that have been explored in the literature include the following.

**(i) 2q14.2 (EN1, SCTR, INHBB) [61]**

The gene *2q14.2* harbours three CpG islands that have been associated with the promoter region of this gene: EN1, SCTR, and INHBB. In a study examining the extent of long-range epigenetic silencing of this gene, at least one of these three CpG islands were methylated in 90% of CRCs. The most commonly methylated promoter region was EN1, with silencing observed in 73% of CRCs and 40% of adenomas. SCTR was also associated with high levels of methylation in carcinoma (53%) and adenoma (33%). Methylation of INHBB was low, detected in only 25% of CRCs and in none of the adenomatous patients. EN1 and INHBB were suggested to be associated with a poorer prognosis in early-stage CRC; however, further investigation was suggested. Bisulfite treatment with melting curve analysis from stool DNA was then used to detect methylated EN1, and a 27% overall sensitivity and 97% specificity were found. The authors concluded that epigenetic suppression of the gene *2q14.2* is frequent in CRC, and hypermethylation was suggested to be a secondary occurrence.

**(ii) ITGA4 [67]**

In a genome-wide search using a microarray-based assay for methylated genes, *ITGA4* was identified in 75% of adenomas of the colon and 92% of CRCs with a diagnostic specificity of 79%. As such, the authors concluded that *ITGA4* is a potential fecal DNA-based early-detection marker for CRC.

**(iii) GATA4 and GATA5 [59]**

The transcriptional factors GATA-4 and GATA-5 are encoded by the genes *GATA4* and *GATA5*, respectively. In the normal body, these transcription factors are essential to the normal development of the gastrointestinal tract and in the evolution of CRC. Hypermethylation of this gene resulting in loss of expression has been documented in primary colorectal, gastric, esophageal, lung, ovarian, and pancreatic cancers. When compared to other genes such as *APC, p14, MGMT, HLTF, p16*, and *RASSFIA*, methylation of *GATA4/5* confers a higher sensitivity and specificity, respectively. *GATA4* methylation is more common than *GATA5. *Fecal DNA from two independent series of CRC patients (*n* = 28 CRC, *n* = 45 control) revealed a *GATA4* sensitivity of 51–71% and a specificity of 84–93%. As such, it was concluded that *GATA4* is a potential molecular marker for CRC screening. Hypermethylation of *GATA4/5* was not significantly associated with tumour-node metastases, stage, tumour location, sex, age at diagnosis, histologic type, or the tumour grade.

**(iv) HIC1 [15]**

The *HIC1* gene encodes the protein hypermethylated-in-cancer-1 (HIC1). The promoter of this gene, localized on chromosome 17p13.3 is often methylated in CRC, but not in aging or normal colonic tissue. Fecal screening for CRC with hypermethylated *HIC1* gene detection compared with FOBT has shown promising results for this epigenetic marker in 26 patients with CRC, 13 with adenoma (≥1 cm), 9 with hyperplastic polyps, 9 with chronic inflammatory bowel disease, and 32 normal-controls. Methylated *HIC1* promoter DNA was not detected in any of the fecal specimens from “normal” or hyperplastic polyps. Specificity was 98%. 42% of CRC-derived samples and 31% from colorectal adenoma were positive for this marker, slightly higher than the sensitivity commonly reported for FOBT.

**(v) miR-34b/c and miR-148a [68]**

MicroRNAs (miRNAs) are noncoding RNAs approximately 22 nucleotides in length responsible for modulation of posttranscriptional activity, targeting mRNA for inhibiting gene expression. *miRNA* genes are believed to play a major role in cancer cell biology, as hypermethylation is thought to drive initiation and progression of polyps towards CRC. Investigation of hypermethylation of miR-34b/c and miR-148a in CRC has been investigated with 5-aza-2′deoxycytidine and MS-PCR. 28 patients with CRC, 12 with high-grade dysplasia, and 39 normal-controls were studied. Hypermethylation of miR-34b/c was identified in 75% of CRC fecal specimens and 16% of high-grade dysplastic polyps. For miR-148a, a trend towards the female sex, an older age, and a decreased overall survival were found associated with these epigenetic changes.

**(vi) OSMR [69]**

Oncostatin M receptor-*β* (*OSMR*) is a receptor for oncostatin M, a member of the interleukin-6 cytokine family that is shown to inhibit a wide variety of carcinomas by inhibiting cellular proliferation. Results have shown that methylation of *OSMR* was detected in the stools of in 26/69 CRC patients (sensitivity of 38%) with a specificity of 95% (77/81). A statistically significant difference was noted between CRC patients and the controls. For Stage II CRC, 56% of stool specimens demonstrated *OSMR* methylation (15/27) and for Stage III 44% (8/18).

**(vii) P16 [54]**

In a study of 45 individuals (25 with CRC and 20 healthy), methylation of *p16* was detected in 20% of patients, with a specificity of 100%.

**(viii) TFPI2 [70]**

Tissue factor pathway inhibitor 2 (TFPI2) is a polypeptide normally able to inhibit Factor Xa and IIa in the coagulation cascade. Using MS-PCR, fecal methylation of this protein's gene promoter was performed, and fluorescent quantitative real-time *Alu* PCR was used to determine the L-DNA quantity. Stool samples were retrieved from patients with CRC (*n* = 60), adenoma (*n* = 20), and healthy-control (*n* = 30). The specificity of this fecal molecular marker was 100%, with a sensitivity of 68.3%. When this fecal marker was combined with L-DNA analysis, a sensitivity of 86.7% was obtained.

**(x) NDRG4 [71]**

*NDRG4* gene on chromosome 16q21–q22.3 encodes for a protein of the same name, N-Myc downstream-regulated gene 4 (NMDRG-4). Unlike many CpG island promoter-methylated genes that are specific for a proximal or a distal tumour, hypermethylation of this gene is present in lesions at both sites. Methylation of the promoter of this gene evidenced in fecal material in patients with CRC (*n* = 75) and normal-control (*n* = 75) was assessed using quantitative MS-PCR. This marker yielded a sensitivity of 61% and specificity of 93%.

**(xi) SFRP-1 [72]**

Hypermethylation of this gene was detected in 29 patients with CRC, 7 with adenoma, and 17 normal-controls using MS-PCR with specially designed primers for methylated/unmethylated promoter sequences of *SFRP1.* This marker yielded an overall sensitivity of 89% and a specificity of 86%, and a significant difference was noted between patients with colorectal neoplasia and the normal-controls.

## 9. Multimarker Panels

With single genetic assays often yielding sensitivities and specificities to premalignant and malignant colorectal lesions lower than anticipated, some research has turned to the development of multimarker panels. To date, no single molecular marker has been identified that is expressed in all CRCs, and the genetic heterogeneity of these lesions suggest that a panel of molecular markers may be better suited for screening purposes [4, 6, 7, 73]. Studies that combine genetic and epigenetic markers have shown a higher sensitivity for colorectal lesions, though often at the expense of specificity [74]. Various combinations of markers have been attempted; however, the number and identity of markers to be included in order to obtain the desired sensitivity and specificity without significantly increasing costs remain undetermined [22]. The commonly studied multimaker panels are discussed below.

### 9.1. PreGen Plus

The best-studied multimarker panel is now commercially available under the name PreGen Plus. PreGen Plus has been available in the United States since 2003 and includes 21 point-mutations on *K-ras * (k12p.1, k12p.2, k12p.3), *APC* (876-2, 1306-2, 1309, 1312-1, 1367, 1378, 1379-3, 1450, 1465-8, 1554), and *p53* (175p.2, 245p.1, 245p.2, 248p.1, 248p.2, 273p.1, 273p.2, 282p.1) combined with BAT-26 and a DNA integrity assay (DIA) to detect abnormalities in the apoptosis pathway and detect L-DNA [23]. This panel has been investigated by several authors, and their results are summarized in Table 3 with a brief discussion below.

**(i) Ahlquist et al. (2000) [32]**

This first multitarget panel studied only 15 markers in *Kras/APC/p53* + BAT-26 and DIA. Freezer-archived fecal samples from 22 patients with CRC, 11 with adenomas (≥1 cm), and 28 normal were retrieved,and sequence-specific hybrid capture was used to isolate human DNA. A sensitivity of 91% for CRC and 82% for adenomas was reported, with a specificity of 93%.

**(ii) Berger et al. (2003) [75]**

These authors used a panel of 19 mutations of *p53, K-ras, *and *APC* and BAT-26 deletions to evaluate the potential of fecal screening. Stool specimens from 100 patients without left-sided neoplasm distal to the splenic flexure were analyzed. 83% of these samples were positive for mutations.

**(iii) Berger et al. (2003) [76]**

This study did not include an assay for L-DNA; however, 19 markers on *p53, APC,* and *KRAS* were tested along with BAT-26 for their mutations in advanced colonic polyps larger than 1 cm in diameter. 28/32 of these polyps demonstrated one or more mutations (sensitivity 88%). Mutations were present in *Kras *(59%), *APC* (33%), and *p53* (22%) but not in BAT-26.

**(iv) Tagore et al. (2003) [23]**

Eighty patients with advanced CRC and 212 controls provided stool samples that were analyzed using this panel of molecular markers. The panel was able to identify 63.5% of invasive CRCs and 57.1% of advanced adenomas, 85.7% of which had high-grade dysplasia. The recorded specificity was 96.2% in patients with no lesions of the colorectum.

**(v) Brand et al. (2004) [36]**

Sixteen patients with CRC provided stool samples on three separate occasions prior to surgical resection. Samples were analyzed for 21 mutations of *p53, APC, *and *K-ras* and deletion of BAT-26. One or more mutations were identified in 11/16 patients (69%) in their first specimen, and there was a 93% concordance between results with positivity in sequential stools in 19/21 patients. A sensitivity of 69% was confirmed on their first specimen, and 86% subsequent stool specimens had identical mutations from the initial, with a 93% overall concordance between initial and subsequent stool analysis. The authors concluded that a single specimen is sufficient for fecal DNA molecular screening.

**(vi) Imperiale et al. (2004) [77]**

4404 participants submitted a stool sample for DNA analysis using this panel, underwent FOBT (Hemoccult II) and a colonoscopy. This molecular marker panel detected 16/31 invasive cancers for a sensitivity of 51.6% and 13/40 with adenoma and high-grade dysplasia for a sensitivity of 32.5%. Overall specificity was 94.4% for this panel. Overall these results were more sensitive than the FOBT in this study.

**(vii) Syngal et al. (2006) [78]**

91 patients with CRC (*n* = 68) and advanced adenomas (*n* = 23) gave stool samples prior to surgery, then 1–3 and 6–9 months after that. The three specimens were analyzed using this 23-marker panel. CRC specimens had a 63% sensitivity, and adenomas a 26%. 47% of all samples had two or more detected gene mutations. *Kras* was the most frequently identified mutation (41%), and distal lesions were more likely than proximal ones to have a positive test. 1–3 months post-op 18% of patients had positive results, and 12/14 were only positive for DIA. Many with these did not have DIA abnormalities prior to treatment, and it was suggested that chemoradiation and surgery may have increased L-DNA. At 6–9 months, only 7% remained positive, suggesting that removal of the neoplastic lesion causes DNA abnormalities to disappear.

**(viii) Berger et al. (2006) [79]**

This study examined the impact this commercial stool screening modality has on increasing adherence to CRC screening recommendations and identification of patients with CRC and adenomas. From 1211 participants, 87% found the specimen collection process easy to perform, and 91% reported that they would likely use the test again. An abnormal stool finding was correlated with the detection of an abnormality at colonoscopy in 49% of patients.

### 9.2. *Vimentin* + DIA

The combination of *Vimentin* methylation and the presence of L-DNA, as detected by DIA, has been examined in the following studies.

**(i) Itzkowitz et al. (2007) [34]**

Forty patients with CRC and 122 normal/controls provided stool samples that had been immediately preserved with a buffer. A gel-based capture was used and analyzed for two separate panels: (a) 22 gene markers with DIA and (b) a combination of *Vimentin* with DIA. The combination (b) yielded the highest results, with an overall sensitivity of 87.5% and a specificity of 82%.

**(ii) Itzkowitz et al. (2008) [8]**

In this follow-up study, 42 patients with CRC and 241 normal-controls were assayed for hypermethylation of *Vimentin* and DIA. *Vimentin* was evaluated with MS-PCR andDIA with real-time PCR. Combined their sensitivity for CRC was 86% and specificity was 82%.

Additional multimarker panels that have been investigated in the literature include the following.

**(iii) *K-ras*, p53, and APC [80]**

Corpocytobiology techniques using colonocytes from fecal matter of 116 CRC patients and 83 normal-controls underwent both cytological examination and DNA analysis. An overall sensitivity of 71% was recorded, with 88% specificity.

**(iv) *K-ras*, APC, Vimentin [81]**

Point mutation on *KRAS, *scanned mutator cluster region of *APC, *and* Vimentin* methylation have been combined to create a molecular marker assay (panel 2) to be tested against the commercial 23-marker PreGen (panel 1) in a multicenter prospective triple-blinded trial. A total of 3764 adults underwent a screening colonoscopy, two FOBTs, and both of these molecular stool panels for analysis. On tissue examination, nearly all specimens analyzed contained at least one marker from the newly proposed panel (#2) whereas less than two-thirds of the tissue specimens contained markers from the commercial panel (#1). Recovery of these molecular markers in stool was nearly equal between the two panels (39% and 40% for panels 2 and 1, resp.). Stool DNA tests were conducted on fecal samples from 69 patients with screen-relevant neoplasia and 54 normal-controls. Panel 2 yielded higher positivity rates than panel 1, 43% versus 20%, including in CRC (58% versus 25%) and adenomas (45% versus 13%).

**(v) *K-ras*, BAT-26, and *TP53* [82]**

Stool DNA from 51 CRC patients was retrieved, and the mutations to *p53, KRAS,* and BAT-26 were identified. The sensitivity for CRC of this panel was 71%.

**(vi) APC, BAT-26, and L-DNA [83]**

57 CRC patients and 44 normal-controls provided stool samples for testing with this panel. Faecal material from 37 patients (65%) contained alterations of at least one of these molecular markers. This panel was found to have 14% higher sensitivity than FOBT.

**(vii) RASSF2, SFRP2 [58]**

The well-studied *SFRP2* was combined with methylation of *RASSF2* due to their high methylation rates in colon and gastric cancers. 788 colorectal and gastric tumour specimens were retrieved as well as 296 fecal specimens from both patients with neoplasia and controls. Extensive methylation of *SFRP2* and *RASSF2* was more common in colorectal tumours, with a sensitivity of 75% among CRC patients and 44% with advanced adenomas. A high false-positivity rate of 10.6% with a specificity of this assay was found to be 89.4%.

**(viii) RARB2, p16, MGMT, and APC [74]**

Promoter methylation of these four genes was analyzed initially in stool samples from 12 patients with newly diagnosed CRC, 20 with colorectal adenomas, and then from an additional 82 patients (20 healthy, 16 with inflammatory bowel disease, 20 with adenomas, and 26 with carcinomas). The first set was analyzed with MS-PCR and the second with methylation-specific melting curve analysis (MS-MCA). The first set yielded a sensitivity of 75% (9/12 patients) with carcinomas and 60% (12/20) with adenomas. In the second set, 62% of carcinomas (16/26) and 40% (8/20) adenomas were detected. The *RARB2* marker was positive in 13% (2/15) of stool samples in patients with inflammatory bowel disease. No methylation was detected in the “normal” group.

**APC, ATM, hMLH1, SFRP2, HLTF, MGMT, GSTP1, and COX-2 [84]**

Fecal samples from patients with CRC (*n* = 20), colorectal polyps (*n* = 30), and normal-controls (*n* = 30) were collected prior to colonoscopy. Using MS-PCR hypermethylation of *APC, ATM, hMLH1, sFRP2, HLTF, MGMT, *and *GSTP1* was analyzed, and COX-2 mRNA was detected using RT-PCR. This combination yielded a sensitivity of 75% in CRC and 68% in adenomas. A specificity of 90% was achieved as 3 normal-control patients were positive for at least one marker. COX-2 was only detected in 50% of cancer and 4% of adenoma patients.

**ITGA4, SFRP2, and p16 [85]**

This combination of markers was used to evaluate fecal specimens from 30 patients with CRC, 25 with adenoma, and 31 healthy-controls. Individually, *ITGA4, SFRP2,* and *p16* were detected in 36.7%, 60.0%, and 40.0% of CRCs and 16.0%, 44.0%, and 24.0% of adenomas, respectively. Combined, this multipanel yielded a sensitivity of 70.0% in CRC and 72.0% in adenoma. This combination yielded an overall specificity of 96.8%.

## 10. Advantages of Stool Testing

The advantages of testing stool samples for molecular markers as a screening test for precancerous and early-stage CRC are multifactorial. These include the following.

### 10.1. Improved Sensitivity and Specificity

The previously-established noninvasive screening technique FOBT and all its subsidiaries rely on the presence of blood derived from a neoplasm in the stool. Comorbidities that may result in this fecal blood, such as active hemorrhoids or an extracolonic lesion, may be confounding [86]. Unlike bleeding that may be intermittent, colonocytes are continually being released and therefore a single specimen is sufficient [22, 73, 87]. Blood invasion is more common in later stages of CRC; however, release of tumour cells into the colonic lumen occurs earlier [7]. Sensitivity and specificity rates of stool DNA testing are higher for many markers [86]. Specificity is enhanced because, unlike serum tests, DNA does not enter the circulation, and heightened sensitivity may result from the large amount of DNA released from CRC compared with normal [22, 73]. This screening may accurately differentiate adenomas with the potential for transformation from purely benign entities. Psychological benefits to both the physician and the patient due to fewer false positives when compared to FOBT may result in improve acceptance and compliance to CRC screening [86].

### 10.2. Complete Screen

Fecal specimen contains genetic material representative of the entire colon, whereas sigmoidoscopy reaches only the distal third [86]. One study found a 20-biomarker panel to be sensitive to 83% of CRCs undetectable by flexible sigmoidoscopy [75] The screening interval may be increased as both cancers and precursor adenomas can be detected [86]. 

### 10.3. Small Amount of Specimen Required

Minute amounts of DNA can be detected and amplified over a billionfold using PCR [73, 87]. These can then be assessed for mutations.

### 10.4. Patient Friendly

Neither dietary nor medication restrictions are required in this screening; therefore, patient compliance is anticipated to increase [22, 81]. The procedure is more patient friendly, as it is noninvasive, requires no cathartic preparation, and does not necessitate an office visit as specimens can be mailed and stored [73, 86]. When in a buffer, stool samples can be transported and stored without degradation [22, 87]. Barriers such as travel, facility capacity, and labour are decreased in the patient-regulated process of specimen collection [8, 73, 86].

### 10.5. Ease of Use

87% of patients in one study found the test easy to perform and 91% indicated they would be willing to take it again in the future [79].

### 10.6. Economical

The overall cost burden of CRC screening may decrease due to a limited number of required colonoscopies. The time interval between these exams may increase, and better identification of patients requiring this invasive screening will be more precise, decreasing the costs of unnecessary colonoscopies [5, 23, 86].

## 11. Disadvantages/Challenges of Stool Testing

The greatest challenge in faecal screening for CRC remains identification and selection of a single or panel of molecular markers with a high sensitivity and specificity. Many targets and combinations of molecular markers have been identified; however, which ones to use and how many of them remain undefined. This is further complicated by the understanding that the more markers used, the higher the cost [86]. Currently, molecular testing of stool specimens is expensive compared to FOBT [4], with the first stool DNA assay to reach market costing $795 [28]. Costs may be further elevated by the labour intensiveness of some of the described methodologies for genetic extraction and analysis [73]. However, cost issues, though very important, are very difficult to resolve. Further, with respect to current noninvasive tests such as FOBT, there is a significant difference in the execution times of the molecular genetic analyses. The dilemma still exists regarding accuracy of such tests versus their costs/benefits for large-scale use; these challenges remain largely unresolved. Such accurate methods are necessary in order to improve sensitivity and specificity. 

Bile salts, hemoglobin degradation products, and complex polysaccharides in faecal specimens may act as inhibitors to PCR, and therefore special techniques are needed to circumvent this complication [58]. Finally, relatively high rates of false-positive and false-negative results limit the accuracy of these tests, thereby restricting their widespread use. False negatives may be due to PCR-based assays not detecting the hypermethylated DNA or due to colonoscopic exam missing a small adenoma. Four potential causes of a false- positive result include (i) experimental conditions of suboptimal standards, (ii) differences in the biology between populations from which normal fecal samples were obtained, (iii) subsistence of low levels of DNA methylation in normal tissue, and (iv) the detection of methylated genes from the normal colon/aberrant crypt foci rather than a relevant lesion [55].

## 12. Future

Detection of precancerous and early-stage CRC is central to improving patient prognosis. Currently available screening techniques have improved their sensitivity to cancerous lesions; however, detection of precursor adenomas with a potential to become malignant remains a challenge. The use of molecular markers in fecal specimens has increasingly become a potential screening tool. Multiple markers have been put forward, some with very promising sensitivity and specificity, yet further validation is required before they can be considered for generalized widespread use. More sensitive PCR strategies and modifications to stool preservation are additional factors that may improve the results of these studies [28]. Once a single/multiple panel of biomarker(s) have been identified, prospective studies with large sample sizes are required for evidence-based statistical assessment. Such tests should include FOBT in parallel for comparative purposes [19]. In the future, such techniques may extend to the detection of supracolonic aerodigestive cancers and aid in the diagnosis of inflammatory bowel disease [30]. Fecal molecular markers have the inherent potential to identify not only malignancies but also benign precursor-malignant entities with a high sensitivity and specificity, thereby coming closer to the ultimate goal of reducing colorectal cancer incidence and mortality [7].

## Figures and Tables

**Table 1:** Studies of *KRAS* genetic alterations in fecal samples.

Authors and reference	Technique	Specimen	Specimen *n* =	Control *n* =	Sensitivity	Specificity
Zhang et al. [39]	Chip-based temperature gradient capillary electrophoresis (TGCE)	CRC	30	15	57%	93%
Chien et al. [40]	RT-PCR + amplified restricted fragment length polymorphism analysis	CRC	29	20	41%	95%
Rengucci et al. [41]	Denaturing gradient gel electrophoresis	CRC	46	18	33%	100%
Notarnicola et al. [42]	PCR amplification and restriction enzyme analysis	CRC	26	None	26.9%	NA
Smith-Ravin et al. [43]	PCR amplification using allele-specific mismatch method	CRC	11	None	50%	NA

**Table 2:** Studies of *SFRP2* genetic alterations in fecal samples.

Authors and references	Technique	Specimen	Specimen *n* =	Control *n* =	Sensitivity	Specificity
Tang et al. [63]	MS-PCR	CRC Adenoma	169 63	30	84% 46%	54%
Wang and Tang [64]	Fluorescence-based RT-PCR	CRC Adenoma	69 34	30	87.0% 61.8%	93.3%
Oberwalder et al. [26]	MethyLight analysis	Adenoma	13	6	46%	100%
Huang et al. [62]	MS-PCR	CRC Adenoma	52 21	24	94.2% 52.4%	95.8%
Müller et al. [65]	MethyLight analysis	CRC	13	13	77%	77%

**Table 3:** Studies of the PreGen panel in the detection of genetic alterations in fecal samples.

Authors and references	Markers	Specimen	Specimen *n* =	Control *n* =	Sensitivity	Specificity
Ahlquist et al. [32]	15 markers: Kras, APC, p53, BAT26, DIA	CRC Adenoma	22 11	28	91% 82%	93%

Berger et al. [75]	19 markers: p523, Kras, APC, BAT26	CRC	100	None	83%	NA

Berger et al. [76]	19 markers: p53, APC, KRAS, BAT26	Polyps ≥1 cm	32	None	88%	NA

Tagore et al. [23]	23 markers: full panel	Advanced CRC	52	212	63.5%	96.2%
		Adenoma	28		57.1%	

Brand [36]	23 markers: full panel	CRC	16	None	69%	NA

Imperiale et al. [77]	23 markers: full panel	CRC Adenoma	31 40	1423	51.6% 32.5%	94.4%

Syngal et al. [78]	23 markers: full panel	CRC Adenoma	68 23	None	63% 26%	NA
